# Compositional and Functional Differences between Microbiota and Cervical Carcinogenesis as Identified by Shotgun Metagenomic Sequencing

**DOI:** 10.3390/cancers11030309

**Published:** 2019-03-05

**Authors:** Minji Kwon, Sang-Soo Seo, Mi Kyung Kim, Dong Ock Lee, Myoung Cheol Lim

**Affiliations:** 1 Division of Cancer Epidemiology and Prevention, National Cancer Center, 323, Ilsan-ro, Ilsandong-gu, Goyang-si 10408, Korea; 74433@ncc.re.kr; 2 Center for Uterine Cancer, National Cancer Center, 323, Ilsan-ro, Ilsandong-gu, Goyang-si 10408, Korea; ssseomd@ncc.re.kr (S.-S.S.); dolee@ncc.re.kr (D.O.L.); mclim@ncc.re.kr (M.C.L.)

**Keywords:** cervical cancer, cervical intraepithelial neoplasia, shotgun metagenomic sequencing, microbiome

## Abstract

Recent studies have reported the potential role of microbiomes in cervical disease. However, little is known about the microbiome composition and function in cervical carcinogenesis. We aimed to identify the compositional and functional alterations of cervical microbiomes in cases of cervical carcinogenesis of Korean women using shotgun metagenomic sequencing. In this study, using shotgun sequencing, we sequenced the cervical metagenomes of cervical intraneoplasia 2/3 (*n* = 17), cervical cancer (*n* = 12), and normal controls (*n* = 18) to identify the microbial abundances and enriched metabolic functions in cervical metagenomes. At the genus level, the microbiota of cervical cancer were differentially enriched with genera *Alkaliphilus*, *Pseudothermotoga*, and *Wolbachia*. Cervical intraepithelial neoplasia (CIN) 2/3 were enriched with *Lactobacillus*, *Staphylococcus,* and *Candidatus Endolissoclinum*. The normal group was enriched with *Pseudoalteromonas* and *Psychrobacter*. Further characterization of the functionalities of the metagenomes may suggest that six Kyoto Encyclopedia of Genes and Genomes (KEGG) orthologies (KOs) that are involved in 10 pathways are associated with an increased risk of CIN2/3 and cervical cancer. Specifically, cervical metagenomes were enriched in the course of peptidoglycan synthesis and depleted by dioxin degradation and 4-oxalocrotonate tautomerase. The Cluster of Orthologous Groups (COG) category ‘Defense mechanisms’ was depleted in cervical cancer patients. Our findings based on shotgun metagenomic sequencing suggest that cervical microbiome community compositions and their metagenomics profiles differed between cervical lesions and normal subjects. Future studies should have larger sample sizes and/or aggregate their results to have sufficient power to detect reproducible and significant associations.

## 1. Introduction

Cervical cancer arises from multi-stage epithelium infection, persistent HPV infection, the progression of persistently HPV-infected epithelium to cervical precursor cells, and invasion through the epithelial basement membrane in the uterine cervix [1]; however, such mechanisms are not sufficient to explain the development of cervical cancer [2]. Various variables such as smoking, oral contraceptive use, sexually transmitted diseases, parity, and dietary factors affect cervical cancer progression with human papillomavirus (HPV) [3]. Recent studies have reported that microbiomes can affect the HPV-related process of cervical carcinogenesis [4]. There is increasing evidence that uterine and vaginal microbiomes play important roles in the carcinogenesis process of the uterine cervix [5,6,7]. The dominance of vaginal microbiota by Lactobacillus species is associated with the maintenance of a healthy reproductive state and relatively low vaginal pH (<4.5) [8]. Bacterial vaginosis (BV) is also associated with cervical intraepithelial neoplasia [9]. BV is characterized by the massive breeding of anaerobic bacteria such as *Gardnerella vaginalis*, *Mobiluncus* species, *Prevotella* species, *Mycoplasma hominis*, and *Atopobium vaginae*, as well as the loss of native Lactobacillus-dominant microflora [10].

In a previous study, we pyrosequenced the 16S rRNA gene and showed that cervical microbial patterns rich in *Atopobium vagiane*, *Lactobacillus iners*, and *Gardnerella vaginalis* and not rich in *Lactobacillus crispatus* were at high risk for cervical intraepithelial neoplasia (CIN) [11,12,13]. However, it is unclear whether cervical cancer and precancerous lesions are associated with alterations in the functional composition of cervical metagenome. Shotgun metagenomic DNA sequencing has provided valuable insight into the phylogeny, biodiversity, metabolic abilities and functional diversity of a variety of organisms [14]. This technology has the potential to provide researchers and clinicians with a better understanding of the pathogenesis of cervical disease and identify unknown pathogens to aid the formulation of both therapeutic and prevention strategies for microbial disease agents [15]. To the best of our knowledge, no study on cervical carcinogenesis has examined the relationship between diagnosis and the compositional or functional alterations of shotgun metagenomes.

In this study, using shotgun sequencing, we sequenced the cervical metagenomes of cervical intraneoplasia 2/3, cervical cancer, and healthy controls to identify microbial abundances and enriched metabolic functions.

## 2. Results

### 2.1. Characteristics of Subjects

The general characteristics of the 47 subjects are provided in Table 1. The study included 18 normal (group A), 17 CIN 2/3 (group B), and 12 cervical cancer (group C) patients. Significant differences were observed for age (*p* = 0.0004): the mean ages of the groups were 45, 41, and 55 years, respectively. No inter-group differences were observed for other variables such as body mass index, oral contraceptive use, smoking, or drinking status.

### 2.2. Taxonomic Characterization of Cervical Microbiome

The majority (73 ± 12%) of the reads were bacterial and dominated by the phyla *Spirochaetes* and *Firmicutes*, representing 60% and 12% of the microbiota, respectively, followed by *Proteobacteria* (8.8%), *Actinobacteria* (4.4%), and *Bacteroidetes* (4.3%) (Supplementary Table S1). The most abundant genera in our cohort were *Leptospira, Gardnerella, Ehrlichia, Lactobacillus, Clostridium*, and *Streptococcus* (Supplementary Figure S1).

### 2.3. Microbial Composition among Normal, Cervical Intraepithelial Neoplasia 2/3, and Cervical Cancer

**Figure 1:**
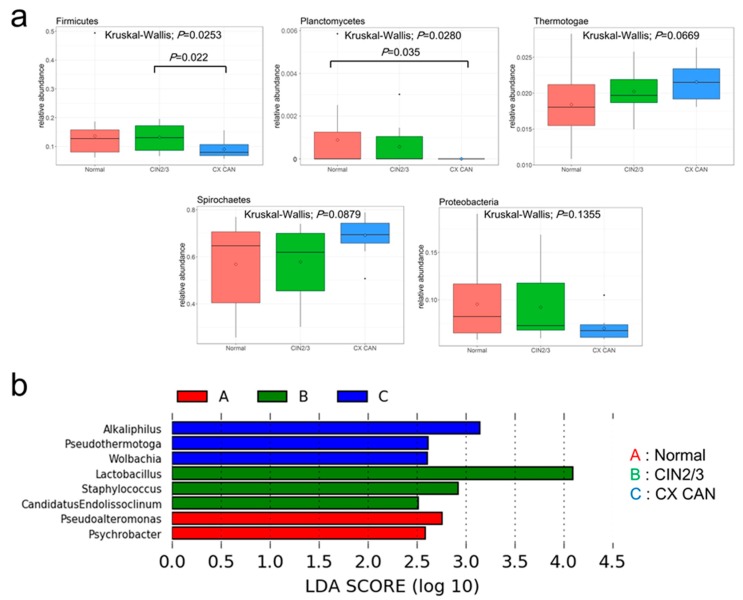
Microbial compositions among normal group, cervical intraepithelial neoplasia 2 or 3, and cervical cancer. (**a**) The differences are in the relative abundances of assembly-based taxonomy for the five major phyla levels. *Firmicute* and *Planctomycetes* showed statistically significant differences (*p* = 0.0253 (B–C: *p* = 0.022); *p* = 0.0280 (A–C: *p* = 0.035)). (**b**) Genus showed statistically significant difference as a result of Linear discriminant analysis (LDA) effect size (LEfSe) analysis. (Logarithmic LDA score >2.5; alpha value <0.05). The post hoc analysis used the Bonferroni method.

The phyla *Firmicutes* and *Planctomycetes* were decreased in cervical cancer patients (*p* < 0.05) relative to the normal or CIN 2/3 group. *Proteobacteria* also was decreased (normal, CIN 2/3 versus cancer, Wilcoxon rank sum test *p* = 0.04), whereas *Spirochaetes* (normal, CIN 2/3 versus cancer, Wilcoxon rank sum test *p* = 0.02) was enriched (Figure 1a). The taxonomic lists that were differentially abundant among the groups were identified by Linear discriminant analysis (LDA) effect size (LEfSe) (minimum LDA score: 2.5) (Figure 1b). At the genus level, the microbiota of cervical cancer were differentially enriched with the genera *Alkaliphilus* (*p* = 0.03), *Pseudothermotoga* (*p* = 0.02), and *Wolbachia* (*p* = 0.01). Meanwhile, CIN 2/3 were enriched with *Lactobacillus* (*p* = 0.02), *Staphylococcus* (*p* = 0.03), and *Candidatus Endolissoclinum* (*p* = 0.01). The normal group was enriched with *Pseudoalteromonas* (*p* = 0.04) and *Psychrobacter* (*p* = 0.03) (Supplementary Figure S2). A principal component analysis revealed that the microbial phyla (and species) abundance did not distinguish the disease groups (healthy controls, CINs, and cervical cancer, Bray *p* = 0.087 and Jaccard *p* = 0.094) (Supplementary Figure S3). Alpha diversity according to the Shannon and Simpson indices did not differ at the phylum or genera level (Supplementary Figure S4).

### 2.4. Distribution and Differences in Relative Abundances of COG Categories

**Figure 2:**
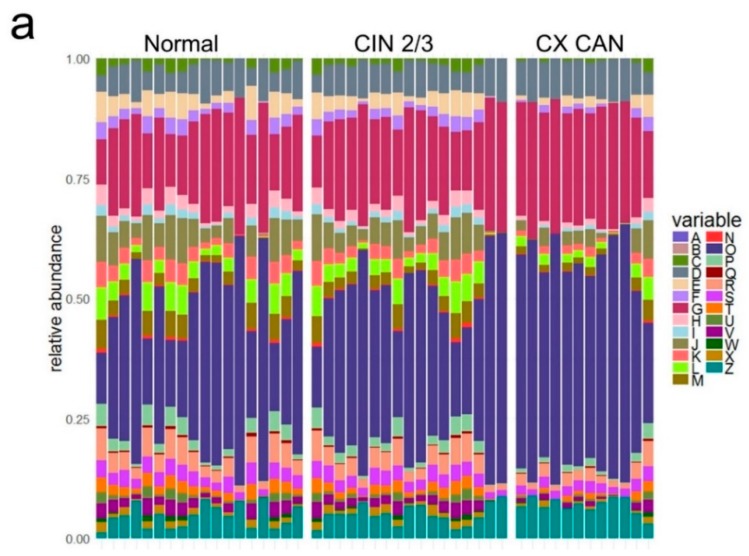

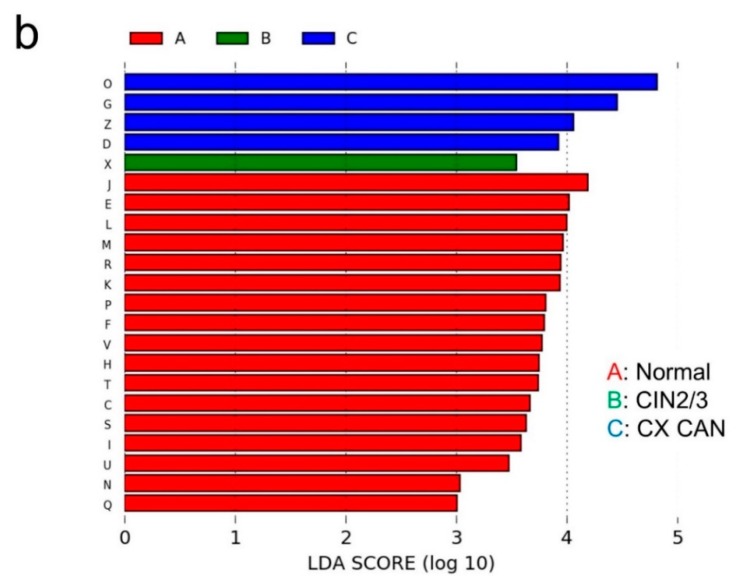
Distribution of relative abundances of Cluster of Orthologous Groups (COG) categories with significant differences in LEfSe analysis. (**a**) Distribution in relative abundances of COG categories. (**b**) Differences in relative abundances of COG categories by LEfSe analysis. (Logarithmic LDA score >2.5; alpha value <0.05). [A] RNA processing and modification; [B] Chromatin structure and dynamics; [C] Energy production and conversion; [D] Cell-cycle control, cell division, chromosome partitioning; [E] Amino acid transport and metabolism; [F] Nucleotide transport and metabolism; [G] Carbohydrate transport and metabolism; [H] Coenzyme transport and metabolism; [I] Lipid transport and metabolism; [J] Translation, ribosomal structure, and biogenesis; [K] Transcription; [L] Replication, recombination, and repair metabolism; [M] Cell-wall/membrane/envelope biogenesis; [N] Cell motility; [O] Post-translational modification, protein turnover, and chaperones; [P] Inorganic ion transport and metabolism; [Q] Secondary metabolites biosynthesis, transport, and catabolism; [R] General function prediction only; [S] Function unknown; [T] Signal transduction mechanisms; [U] Intracellular trafficking, secretion, and vesicular transport; [V] Defense mechanisms; [W] Extracellular structures; [X] Mobilome: prophages, transposons; [Z] Cytoskeleton.

To obtain insight into the functional properties of cervical microbes, a significant detection gene was assigned to the Cluster of Orthologous Groups (COG) database [16] for microbial function analysis. The major COG categories detected in our subjects belonged to the functional categories “Cellular processes and signaling” (mainly ([D] cell-cycle control, cell division, chromosome partitioning, [O] post-translational modification, protein turnover, and chaperones, [Z] Cytoskeleton) and “Metabolism” (mainly ([G] Carbohydrate transport and metabolism) (Figure 2a, Supplementary Table S2). The COG categories that were differentially abundant among the groups were identified by LEfSe. Of the 25 COG categories, 22 showed an LEfSe LDA score of 2.5 or higher (Figure 2b). The COG categories of [V] Defense mechanisms, [T] Signal transduction mechanisms, and [K] Transcription were depleted in cervical cancer patients. Meanwhile, the dominant categories “Cellular processes and signaling” ([D] cell-cycle control, cell division, chromosome partitioning, [O] post-translational modification, protein turnover, chaperones, and [Z] Cytoskeleton) and “Metabolism” ([G] Carbohydrate transport and metabolism) were enriched in cervical cancer patients. The 17 COG categories with lesser abundances were significantly enriched in the normal controls.

### 2.5. Metabolic Functions of Cervical Microbiota

**Figure 3:**
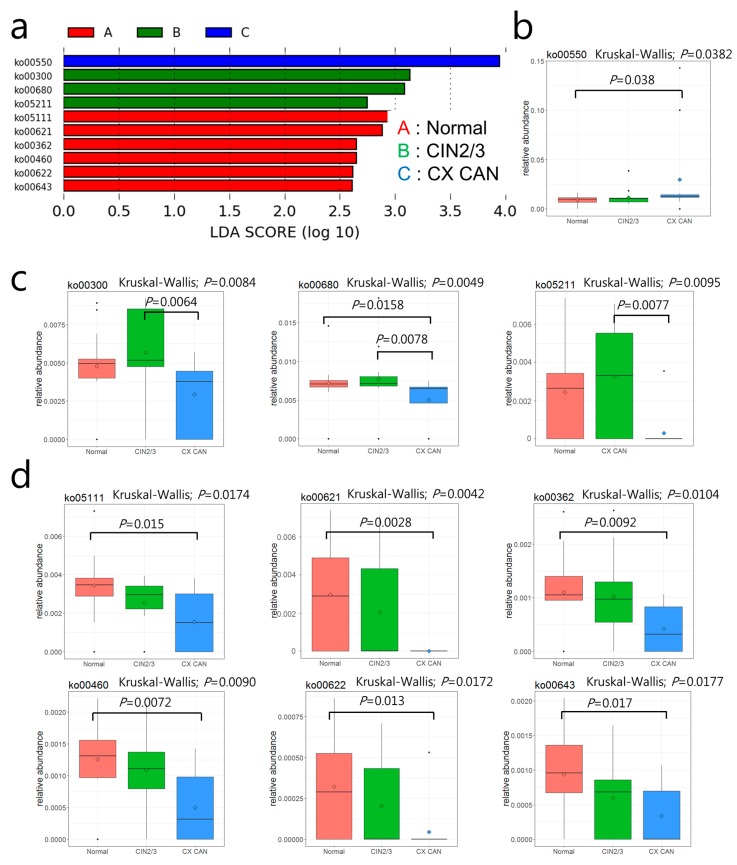
Differences in Kyoto Encyclopedia of Genes and Genomes (KEGG) pathway profiles among groups. (**a**) LEfSe results of KEGG pathway. (**b**) Relative abundances of ko00550 were significantly enriched in cervical cancer. (**c**) KEGG pathways were significantly enriched in CIN 2 or 3. (**d**) KEGG pathways were enriched in normal subjects (logarithmic LDA score >2.5; alpha value <0.05). The post hoc analysis used the Bonferroni method.

We conducted a LEfSe analysis to distinguish among the disease groups and 224 Kyoto Encyclopedia of Genes and Genomes (KEGG) pathways. Ten KEGG pathways were presented significantly differently among the groups (Figure 3a): six pathways were enriched in the cervical metagenomes of the controls, while three pathways and one pathway were enriched in the CIN2/3 and cancer subjects, respectively (*Kruskal–Wallis* test). Of the 2860 KEGG orthologies (KOs), 118 were found to be statistically significant in the LEfSe analysis (Supplementary Figure S5). Of the 118 KOs, six belonging to 10 KEGG pathways were observed (Table 2).

The KEGG pathway of peptidoglycan biosynthesis (ko00550) was enriched in cervical cancer subjects (*p* = 0.03) (Figure 3b), while ko00300 (Lysine biosynthesis, *p* = 0.01), ko00680 (Methane metabolism, *p* = 0.005), and ko05211 (Renal cell carcinoma, *p* = 0.01) were enriched in CIN2/3 (adj. *p* < 0.05, Figure 3c). We also found that the pathways of Biofilm formation in Vibrio cholerae (ko05111, *p* = 0.01), dioxin degradation (ko00621, *p* = 0.004), benzoate degradation (ko00362, *p* = 0.01), cyanoamino acid metabolism (ko00460, *p* = 0.09), xylene degradation (ko00622, *p* = 0.01), and styrene degradation (ko00643, *p* = 0.01) were abundant in the normal controls.

**Figure 4:**
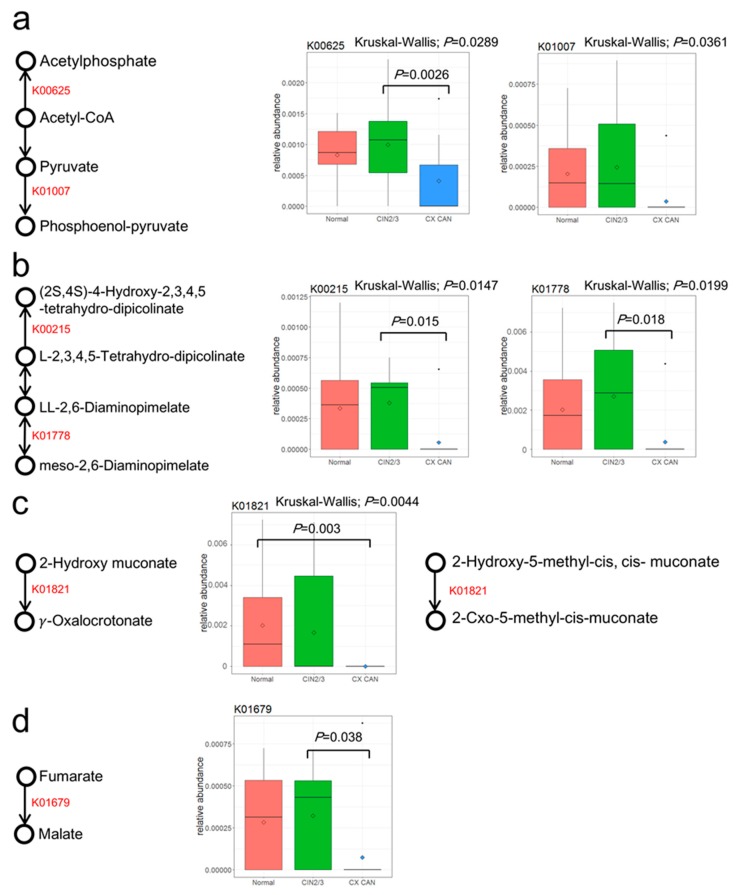
Differences among KEGG orthologies (KOs). (**a**) KOs are associated with ko00680. K00625 [phosphate acetyltransferase] and K01007 [pyruvate, water dikinase] enriched in CIN 2 or 3 (*p* = 0.02893 (B–C: *p* = 0.0026); *p* = 0.0361). (**b**) KOs are associated with ko00300. K00215 [4-hydroxy-tetrahydrodipicolinate reductase] and K01778 [diaminopimelate epimerase] enriched in CIN 2 or 3 (*p* = 0.0147 (B–C: *p* = 0.015); *p* = 0.0199 (B–C: *p* = 0.018)). (**c**) K01679 is associated with ko05211. K01679 [fumarate hydratase, class II] enriched in CIN 2 or 3 (*p* = 0.0318 (B–C: *p* = 0.038)). (**d**) K01821 [4-oxalocrotonate tautomerase] is associated with ko00621, ko00362, and ko00622. K01821 enriched in normal subjects (*p* = 0.0044 (A–C: *p* = 0.003)). The post hoc analysis used the Bonferroni method.

Several metabolites were involved in these KEGG pathways (Table 2): K00625 (phosphate acetyltransferase, Figure 4a) and K01007 (pyruvate, water dikinase), which are involved in ko00680 (methane metabolism) were decreased in cervical cancer, as were K00215 (4-hydroxy-tetrahydrodipicolinate reductase, Figure 4b) and K01778 (diaminopimelate epimerase), which are involved in ko00300 (lysine biosynthesis), and K01679 (fumarate hydratase, class II, Figure 4c), which is involved in Ko05211 (renal cell carcinoma). Consequently, we also found that a lower abundance of K01821 (4-oxalocrotonate tautomerase, Figure 4d) in cervical cancer was involved in the pathways of K01821 (4-oxalocrotonate tautomerase, Figure 4d) in ko00621 (dioxin degradation), Ko00362 (Benzoate degradation), and Ko00622 (cyanoamino acid metabolism). A principal component analysis showed that the abundances of the KEGG pathway by group (Supplementary Figure S6) did not distinguish the disease groups (controls, CIN 2/3, and cervical cancer).

## 3. Discussion

This study performed shotgun metagenomic sequencing of cervical-swab samples from Korean women. Thereby, we observed significant microbiome profile differences and functionalities among a normal group, CIN 2/3, and cervical cancer. An LEfSe analysis showed statistically significant differences among eight genuses and 10 pathways. Further, six KOs within those pathways were found. The COG category ‘Defense mechanisms’ was depleted in cervical cancer patients. Furthermore, this category has been reported to be deficient in ileal Crohn’s disease as compared with healthy controls [17].

Bacteria, as a key player, were increased in number in the initiation and progression of a number of malignant tumors; *Fusobacteria* has been implicated for its role as a possible pro-carcinogenic bacterial phylum. Our findings showed that *Alkaliphilus*, *Pseudothermotoga*, and *Wolbachia* were statistically significantly enriched in cervical cancer. *Alkaliphilus* has been reported to be more numerous in a hepatic encephalopathy negative group [18]. *Wolbachia* has been reported for its immunological role in filarial infection, especially in humans [19]. In the present study, *Lactobacillus, Staphylococcus*, and *Candidatus Endolissoclinum* were found in CIN 2 or 3. *Lactobacillus* is a genus containing *L. crispatus* and *L. iners*. *L. crispatus* is predominant in healthy females, and *L. iners* is predominant in unhealthy individuals [11,20]. *Staphylococcus* spp. has been associated with colon carcinoma, catheter-related bloodstream infections, and neutropenic patients [21,22,23]. In the current study, the normal group were found to have been enriched with the genuses *Pseudoalteromonas* and *Psychrobacter*. *Pseudoalteromonas* has been reported to be lower in Helicobacter pylori-infected patients [24], and *Psychrobacter* was reduced in colorectal carcinoma patients [25]. However, the associations of *Pseudothermotoga* and *Candidatus Endolissoclinum* with human diseases is not yet clear.

Among our results, the alpha diversity Shannon and Simpson indices were found to be lower in women with cervical cancer, but these findings were without statistical significance. This result is consistent with previous studies showing no difference between healthy normal cases and precancerous lesions of the cervix [5,20]. Other results have shown that Lactobacillus with low bacterial diversity and low pH is dominant in the healthy vagina, whereas in the unhealthy vagina, it shows high bacterial diversity with low counts of lactobacilli and high counts of anaerobic bacteria [26]. This difference seems to be due to the sequencing method employed [15,27].

In the KEGG pathway, peptidoglycan biosynthesis is enriched in cases of cervical cancer. Peptidoglycan biosynthesis is also enriched in patients with symptomatic atherosclerosis [28], which indicates that the increased production of peptidoglycan from gut microbiota improves neutrophil functionality and the innate immune system, thus leading to symptomatic atherosclerosis. Inflammation has been identified as an important contributor to the pathogenesis of atherosclerosis. Our results suggest that the cervical metagenome might contribute to the development of cervical cancer and precancerous lesions by acting as a regulator of host inflammatory pathways. A bacterial cell-wall polymer, peptidoglycan, in its maintenance of cell integrity against osmotic pressure, is essential for cell survival [29].

Among the other results of the present study, lysine biosynthesis, methane metabolism, and renal cell carcinoma were enriched in CIN 2 or 3. Lysine biosynthesis has been found to be diminished in ulcerative colitis and Crohn’s disease [30]. Lysine is an amino acid that is essential for humans, and is synthesized in bacteria via the diaminopimelate pathway, which acts in the first and second enzymatic reactions in the biosynthesis of isoleucine, methionine, and threonine. In these amino acids’ biosynthesis, the initial enzymatic step is catalyzed by aspartokinase [31]. Methane metabolism is one of the major metabolic processes of colorectal carcinoma [32]. Methane, as one of the end products of fermentation in the gastrointestinal system, along with fermented gases including hydrogen, readily appears in breath [33]. Methane metabolism by bacteria in the large intestine has been reported as early as 1977. This report showed that the excretion of methane in breath occurred twice as frequently in patients with colonic cancer as in normal individuals [34]. This suggests a difference in the anaerobic intestinal flora between patients and normal subjects, and implies that colorectal cancer can be caused by carcinogens formed by anaerobic bacteria’s dehydrogenation of nuclear bile acid in the colon [35]. It is possible that these and other functional roles are related to cervical cancer, but further functional studies are needed before any firm conclusions can be drawn. Renal cell carcinoma is one of the significantly down-regulated pathways related to Parkinson’s disease [36]. Meanwhile, some other pathways related to human diseases also have been identified as down-regulated in Parkinson’s disease. The most important finding was that several cancer-related pathways were uncovered, including colorectal cancer, renal cell carcinoma, and endometrial cancer. This is consistent with previous reports of low cancer rates among patients with Parkinson’s disease. It has been suggested that the high levels of total body potassium in Parkinson’s patients might be a protective factor against cancer [37].

Biofilm formation in Vibrio cholerae, dioxin degradation, styrene degradation, benzoate degradation, xylene degradation, and cyanoamino acid metabolism were enriched in the present study’s normal subjects. Biofilm formation in Vibrio cholerae, correspondingly, has been identified in associated pathways in Type 2 diabetes (T2D) patients [38]. Particularly, in an independent comparative study of the microbiomes of European T2D patients [38], cysteine and methionine metabolism was one of the most significant T2D-related pathways identified. A significant association with T2D only when applying a modified pipeline also has been found in biofilm formation in Vibrio cholera, both of which had high levels of blood sugar. These results suggest that identifying functional variation that is often masked by standard metagenomic bioinformatic processing can improve our ability to identify important associations between microbiome functionality and disease. A loss of the vitamin D receptor in rats has been reported to reduce the dioxin degradation pathway [39]. Benzoate degradation and styrene degradation were reported in Helicobacter pylori, which is associated with gastric cancer [40]. Xylene degradation [41] as well as polycyclic aromatic hydrocarbon and xylene degradation, have been found to be abundant in current smokers. These chemicals are components of cigarette smoke [42], and thus alterations in the ability to break down these substances can have toxic consequences for the host. It is surprising that some of the xenobiotic degradation pathways are depleted by smokers, due to the need for the bacterial up-regulation of these pathways to detoxify tobacco smoke. Cyanoamino acid metabolism, for instance, appears to be up-regulated in stage II rectal carcinoma tissues [43].

Our study has several strengths. First, the use of whole-genome shotgun metagenomic sequencing for microbiome analysis allowed us to not only comprehensively study overall bacterial community composition and specific oral taxon abundances, but also perform a functional analysis of the COG and KEGG pathways. Analysis using shotgun metagenomic sequencing can find and identify unknown microbiota that can play a role in the process of cervical carcinogenesis. It has been reported that there are differences in 16S rRNA sequencing and whole-genome shotgun metagenomic sequencing [15,27]. 16S rRNA sequencing can be biased due to unequal amplification of the 16S rRNA gene in species, although shotgun metagenomic sequencing might not be deep enough to detect the 16S rRNA gene of rare species. Second, in this study, we examined the compositional and functional alterations of the cervical microbiomes in cervical carcinogenesis for normal group, CINs as precancerous lesions, and cervical cancer. The present report is, as far as we know, the first to have been based on the utilization of shotgun metagenomic sequencing to study the compositional and functional alterations of cervical microbiomes in cervical carcinogenesis. However, the study on which the report is based was limited by an insufficient sample size for determination of the association of cervical metagenomes with cervical carcinogenesis. Further studies that boast larger sample sizes or pooled cross-study data and that are reproducible and have sufficient power to detect significant associations are needed. HPV infection status, HPV genotype, and sexual activity were not considered as potential variables in this study, and their exclusion could have biased the results [44]. Finally, the case-control studies were limited to one point in time in order to prevent the direction of a series of events. Moreover, case-control studies are inherently limited in their causality–inference utility.

## 4. Materials and Methods

### 4.1. Study Design and Subjects

From March 2006 to the present, the Korean HPV cohort study including women aged 18–65 years has been ongoing. Details on the cohort design criteria are available in a previous paper [45]. The subjects enrolled in the study were patients who had been given a diagnosis of CIN or cervical cancer that was histologically proven. Subjects receiving any therapy or surgery or using immunosuppressive agents were excluded at enrollment. Detailed self-administered health and lifestyle questionnaires, including questions on behavior related to alcohol consumption, were completed at enrollment. From the patients with CIN 2/3 or cervical cancer, a cervical swab was obtained at the first visit prior to any treatment such as Large Loop Excision of the Transformation Zone (LLETZ), surgical or radiation therapy, or chemotherapy. Cervical swabs were collected for Papanicolaou smear tests and tumor HPV DNA tests using a Cervical Sampler Brush (Cervical Sampler, Digene Co., MD, USA). A total of 47 women were randomly selected from 18 normal subjects, 17 CIN 2 or 3 subjects, and 12 cervical cancer patients. All of the study participants consented in writing according to the requirements of the Institutional Review Board. This study was approved by the Institutional Ethics Committee of the National Cancer Center of Korea (IRB No. NCC2016-0147).

### 4.2. DNA Extraction, Sequencing, and Quality Check

Metagenomic DNA samples were extracted using the FastDNA Spin extraction kit (MP Biomedicals, Santa Ana, CA, USA). All of the samples were sequenced by Illumina HiSeq2500 at Chunlab, Inc. in Seoul, Korea. Paired-end reads were generated with 250 bp. The average insert size of the libraries was 350 bp.

After sequencing, FastQC [46] was used to check the read quality. Since the raw data includes host (human) genome and adapter sequences, raw sequence reads were filtered and trimmed with KneadData [47] in order to reduce biases in the analysis. The Trimmomatic [48] option in KneadData was used with ‘ILLUMINACLIP:TruSeq3-PE-2.fa:2:30:10:8:true LEADING:3 TRAILING:3 SLIDINGWINDOW:4:20 MINLEN:36’. The analysis was carried out using the non-host, non-adapter sequence.

### 4.3. Taxonomical Analysis

K-mer-based taxonomical assignment was performed with CLAssifier based on Reduced K-mers (CLARK) [49]. For each sample, all of the reads were classified using the National Center for Biotechnology Information (NCBI) RefSeq database (bacteria and viruses) from phylum to species. The classification algorithm was based on discriminative k-mers, and the classification was performed with the parameter ‘-m 0 (full mode)’. After classification, the abundance was estimated using the count, and the proportion of each target was identified using CLARK with the given parameter: ‘--highconfidence’.

### 4.4. De Novo Assembly and Gene Prediction

De novo assembly was performed for each sample with IDBA-UD [50]. Based on the de Bruijn graph approach, the optimal result was selected from among the multiple k-mers (73, 83, 93, and 101). The assembled contigs were classified with Fragment Classification Package (FCP) [51] using the NCBI RefSeq and taxonomy databases. First, classification with Naïve Bayes (NB) was performed. Next, classification with Epsilon-NB was performed, after which the numbers of fragments and base pairs assigned to the different taxonomic categories were calculated.

Finding genes and assigning their functions are two of the main purposes of shotgun metagenomic sequencing. Gene prediction was performed with Prodigal [52] in the metagenomics mode. After gene prediction, the obtained genes were searched against the COG [16] databases using Basic Local Alignment Search Tool (BLAST) [53] with an *E*-value threshold of 1 × 10^−5^, in order to carry out functional annotation.

Finally, to identify which pathways the predicted genes are involved in, the KEGG [54,55,56] database was used with BLAST under the same conditions. Bacteria and virus sequences from the KEGG database were used for the analysis. Also, the number of genes matched for each KO were found using an in-house script and compared by sample.

### 4.5. Statistical Analysis

Differences in the demographic and clinical characteristic of the participants were subjected to the *Kruskal–Wallis* test for continuous variables. Categorical variables were analyzed by the *chi-squared* test, and Fisher’s exact test was performed when more than 25% of the cells with an expected frequency of less than five were used. The alpha diversities of the Shannon and Simpson indices were calculated for each group. Beta diversity was calculated with principal coordinates analysis (PCoA) according to the Bray-Curtis and Jaccard distances. A permutational multivariate analysis of variance (PERMANOVA) was implemented to determine significance in distance. Diversity and the PERMANOVA results were analyzed using the R package’s “vegan” [57]. To determine the taxonomic and genetic features that were differentially abundant, LEfSe was performed with alpha = 0.5 and an LDA score = 2.5 as the standard [58]. The post hoc analysis used the Bonferroni method with the R package’s “PMCMR” [59]. The visualization used the ggplot2 package [60]. The analysis was performed with SAS 9.4 (SAS Institute, Cary, NC, USA) and the R platform (version 3.4.3) (The R Foundation for Statistical Computing, Vienna, Austria).

## 5. Conclusions

In summary, in this case-control study of cervical metagenomics using shotgun metagenomic sequencing, we observed significantly different microbial abundance and enriched metabolic functions in cervical metagenomes between normal, CIN 2/3, and cervical cancer. At the genus level, the microbiota of cervical cancer was differentially enriched with the genera *Alkaliphilus*, *Pseudothermotoga,* and *Wolbachia*; CIN 2/3 were enriched with *Lactobacillus, Staphylococcus*, and *Candidatus Endolissoclinum*, and the normal group was enriched with *Pseudoalteromonas* and *Psychrobacter*. Further characterization of the functional capacities of the metagenomes revealed that six KOs involved in 10 pathways are associated with an increased risk of CIN2/3 and cervical cancer. Specifically, cervical metagenomes were enriched in peptidoglycan synthesis and depleted in dioxin degradation and 4-oxalocrotonate tautomerase. The COG category of ‘Defense mechanisms’ was depleted in cervical cancer patients. This is the first study using shotgun metagenomic sequencing for the cervical carcinogenesis stage. Even though our study cannot provide evidence of direct causation, these findings demonstrate differences in cervical microbiomes and their functional bacteria-involved pathways among normal, CIN 2 or 3, and cervical cancer patients.

## Figures and Tables

**Table 1:** Characteristics of Normal, Cervical Intraepithelial Neoplasia (CIN) 2/3, and Cervical Cancer Subjects.

Characteristics	Normal (Group A, *n* = 18)	CIN 2/3 (Group B, *n* = 17)	Cervical Cancer (Group C, *n* = 12)	*p* ^a^
Age (year) ^b^	45.6 (7.72)	41.1 (6.98)	55.9 (10)	0.0004
BMI (kg/m^2^) ^b^	21.3 (2.66)	21.3 (2.56)	22.9 (4.03)	0.4852
Energy intake (kcal/day) ^b^	1892 (367)	1839 (435)	1953 (452)	0.8232
HPV ^c^	11 (61.1)	12 (80)	8 (72.7)	0.5435
Post-menopausal ^c^	7 (41.2)	3 (18.7)	7 (63.6)	0.0604
Oral contraceptive use (Yes&ever) ^c^	3 (16.7)	5 (29.4)	4 (33.3)	0.5343
Hormone treatment (Yes&ever) ^c^	4 (23.6)	0 (0)	1 (9.1)	0.1552
Current smoker ^c^	2 (11.8)	2 (12.5)	1 (9.1)	1.0
Passive smoker ^c^	10 (58.8)	13 (81.3)	7 (63.6)	0.3587
Alcohol drinker ^c^	12 (70.6)	14 (87.5)	6 (54.5)	0.0615

^a^*p* value calculated by *chi-squared* test for categorical variables and by *Kruskal–Wallis* test for continuous variables. Fisher’s exact test was performed when the categorical variable was more than 25% of the cells with an expected frequency of five or less. ^b^ Mean (standard deviation) ^c^ *n* (%) BMI: body mass index; HPV: human papillomavirus.

**Table 2:** Mean relative abundance of 10 KEGG pathways and six KEGG orthologies (KOs).

KEGG Pathway	Pathway Name	Normal	CIN2/3	Cervical Cancer	*p* ^a^	KEGG Orthology	KO Name	Normal	CIN2/3	Cervical Cancer	*p* ^a^
ko00550	Peptidoglycan biosynthesis	0.8881	1.1465	2.9481	0.0382						
ko00300	Lysine biosynthesis	0.4785	0.5669	0.2940	0.0084	K00215	4-Hydroxy-tetrahydrodipicolinate reductase	0.0334	0.0380	0.0055	0.0146
						K01778	Diaminopimelate epimerase	0.0202	0.0271	0.0036	0.0199
ko00680	Methane metabolism	0.7161	0.7716	0.4996	0.0049	K00625	Phosphate acetyltransferase	0.0831	0.0997	0.0407	0.0289
						K01007	Pyruvate, water dikinase	0.0203	0.0243	0.0036	0.0361
ko05211	Renal cell carcinoma	0.0244	0.0327	0.0030	0.0095	K01679	Fumarate hydratase, class II	0.0284	0.0322	0.0073	0.0318
ko05111	Biofilm formation in Vibrio cholerae	0.3439	0.2532	0.1552	0.0174						
ko00621	Dioxin degradation	0.0296	0.0204	0	0.0042	K01821	4-Oxalocrotonate tautomerase	0.0202	0.0167	0	0.0044
ko00362	Benzoate degradation	0.1100	0.1027	0.0421	0.0104	K01821	4-Oxalocrotonate tautomerase	0.0202	0.0167	0	0.0044
ko00460	Cyanoamino acid metabolism	0.1251	0.1093	0.0502	0.0090						
ko00622	Xylene degradation	0.0320	0.0204	0.0044	0.0172	K01821	4-Oxalocrotonate tautomerase	0.0202	0.0167	0	0.0044
ko00643	Styrene degradation	0.0939	0.0596	0.0339	0.0177						

^a^*p* value calculated by *Kruskal–Wallis* test.

## Supplementary Materials

The following are available online at https://www.mdpi.com/2072-6694/11/3/309/s1, Figure S1: Box plots of relative abundances of major genuses, Figure S2: Differences among genus level, Figure S3: PCoA in phylum, Figure S4: Alpha indices in genera, Figure S5: Comparison of KOs using LEfSe to identify differentially abundant KEGG pathways, Figure S6: PCoA in KEGG pathway, Table S1: Mean cervical microbial abundances in 31 phyla, Table S2: Mean relative abundances of COG categories.

## Abbreviations

CIN	cervical intraepithelial neoplasia
LEfSe	Linear discriminant analysis (LDA) effect size
COG	Cluster of Orthologous Groups
KEGG	Kyoto Encyclopedia of Genes and Genomes
PCoA	principal coordinates analysis
PERMANOVA	Permutational multivariate analysis of variance
Kos	KEGG orthologies
LLETZ	Large Loop Excision of the Transformation Zone
