# Supplementary material for: Compositional and Functional Differences between Microbiota and Cervical Carcinogenesis as Identified by Shotgun Metagenomic Sequencing
Source: Cancers (Basel). 2019 Mar 5;11(3):309. doi: 10.3390/cancers11030309 (PMC6468638; doi:10.3390/cancers11030309)
Supplement: Supplementary file 1 [file cancers-11-00309-s001.pdf]

Supplementary Materials: Compositional and Functional Differences between Microbiota and Cervical Carcinogenesis as Identified by Shotgun Metagenomic Sequencing

Minji Kwon, Sang-Soo Seo, Mi Kyung Kim, Dong Ock Lee and Myoung Cheol Lim

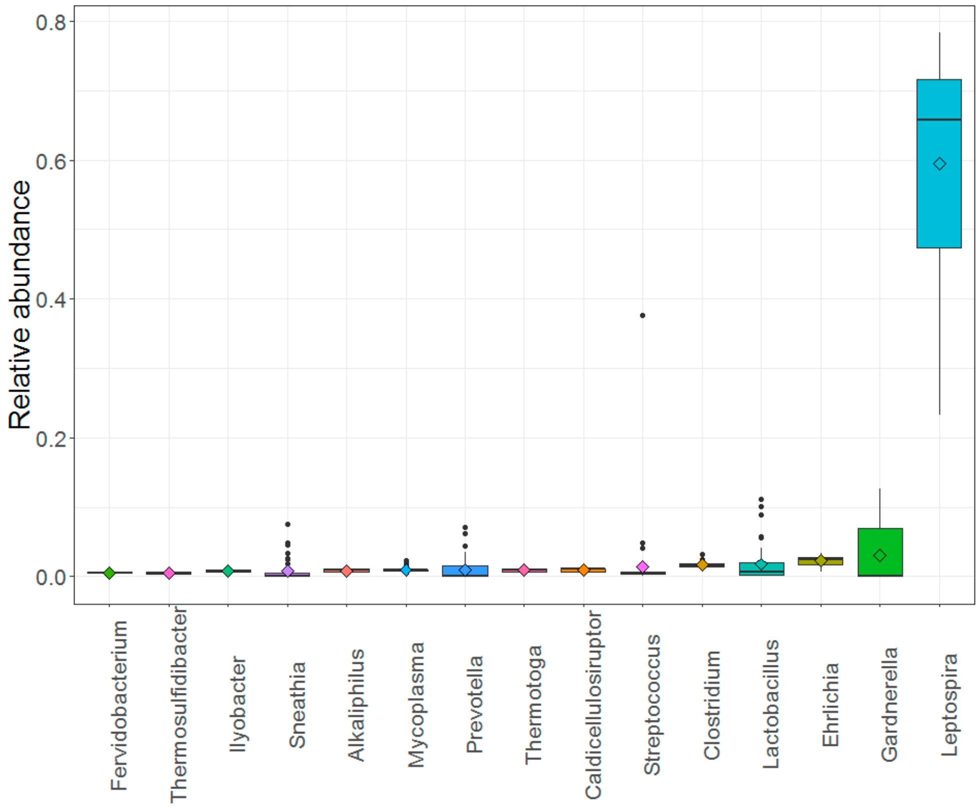

Figure S1. Box plots of relative abundances of major genera.

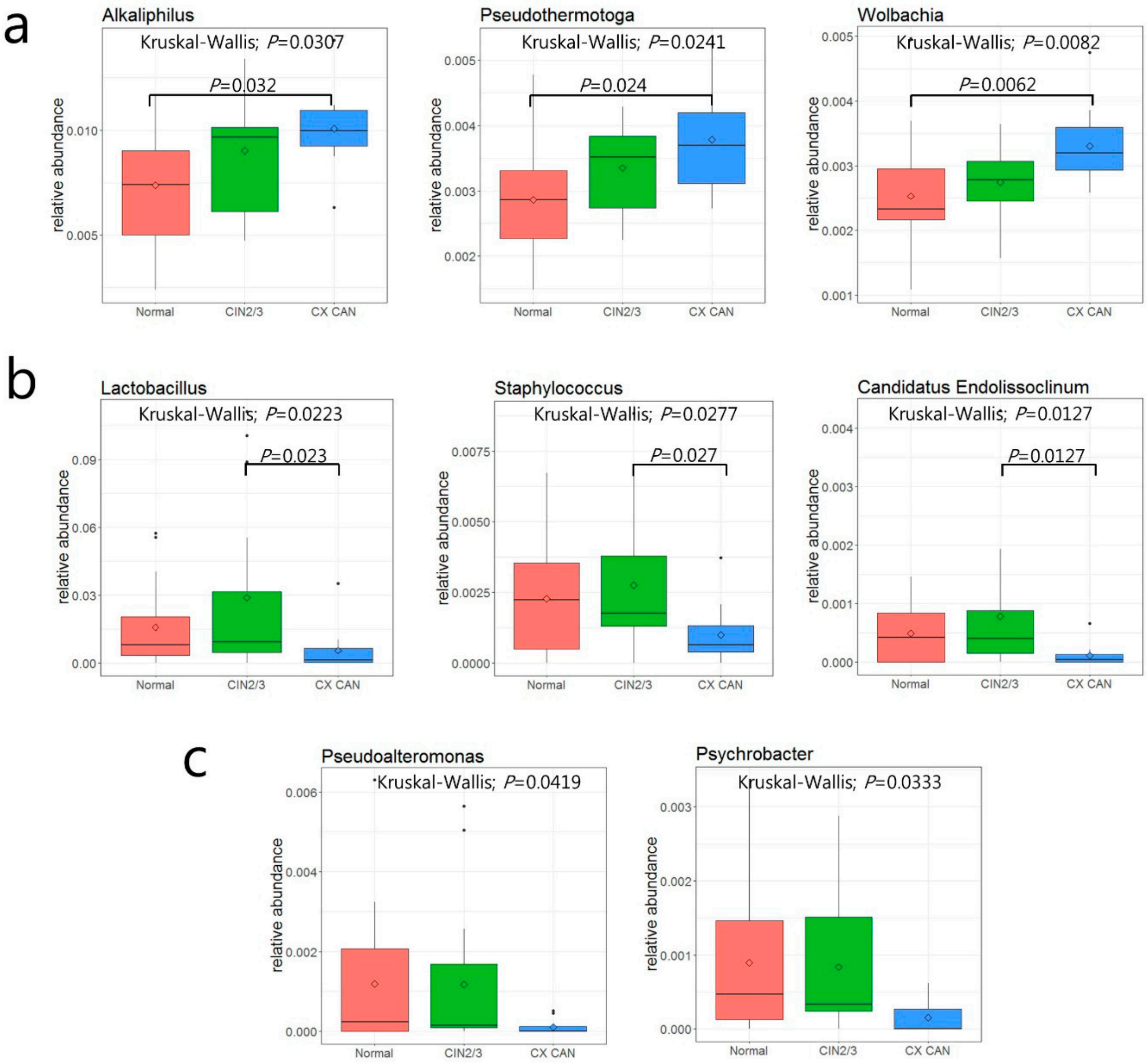

Figure S2. Differences among genus level. Kruskal-Wallis test was performed to test abundances of significant LEfSe-result genera among groups. (**a**) Cancer enriched. *Alkaliphilus* (p = 0.0307; A–C: p = 0.032); *Pseudothermotoga* (p = 0.0241; A–C: p = 0.024); *Wolbachia* (p = 0.0082; A–C: p = 0.0062). (**b**) *Lactobacillus* (p = 0.0223; B–C: p = 0.023); *Staphylococcus* (p = 0.0277; B–C: p = 0.027); *Candidatus Endolissoclinum* (p = 0.0127; B–C: p = 0.011). (**c**) Normal enriched. *Pseudoalteromonas* (p = 0.0419; B-C: p = 0.056); *Psychrobacter* (p = 0.0333; A–C: p = 0.055; B–C p = 0.065); The post-hoc analysis used the Bonferroni method. A: Normal B: CIN2/3 C: CX CAN.

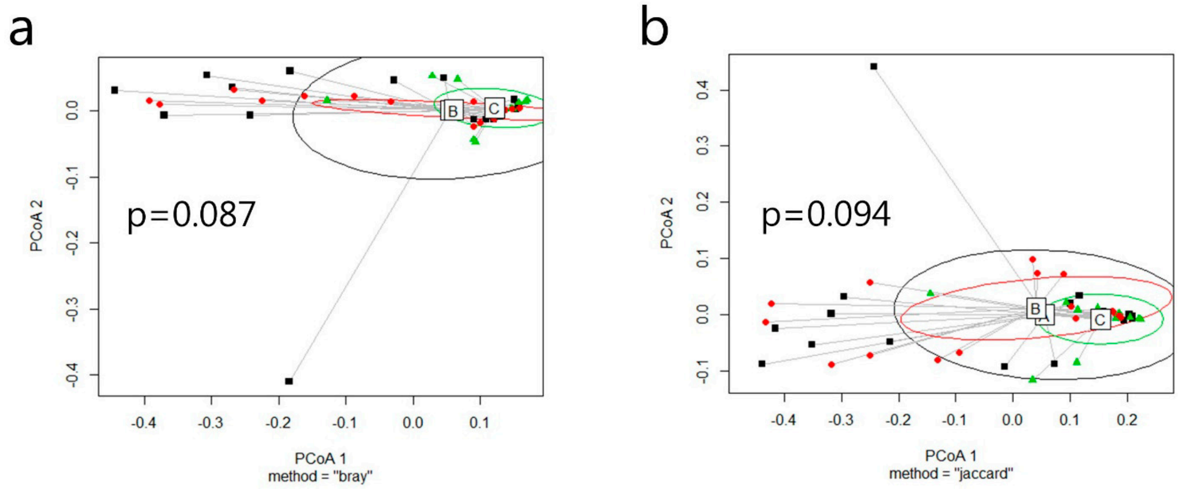

Figure S3. PCoA in phylum. (**a**) Bray-Curtis p = 0.087, (**b**) Jaccard p = 0.094. A: Normal, B: CIN 2/3, C: CX CAN.

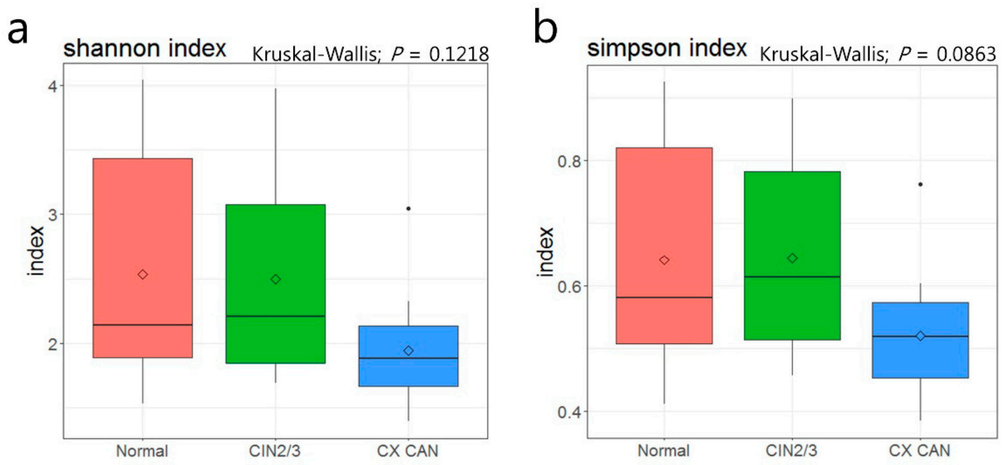

Figure S4. Alpha indices in genera. (**a**) Shannon index p = 0.1218, (**b**) Simpson index p = 0.0863. A: Normal, B: CIN 2/3, C: CX CAN.

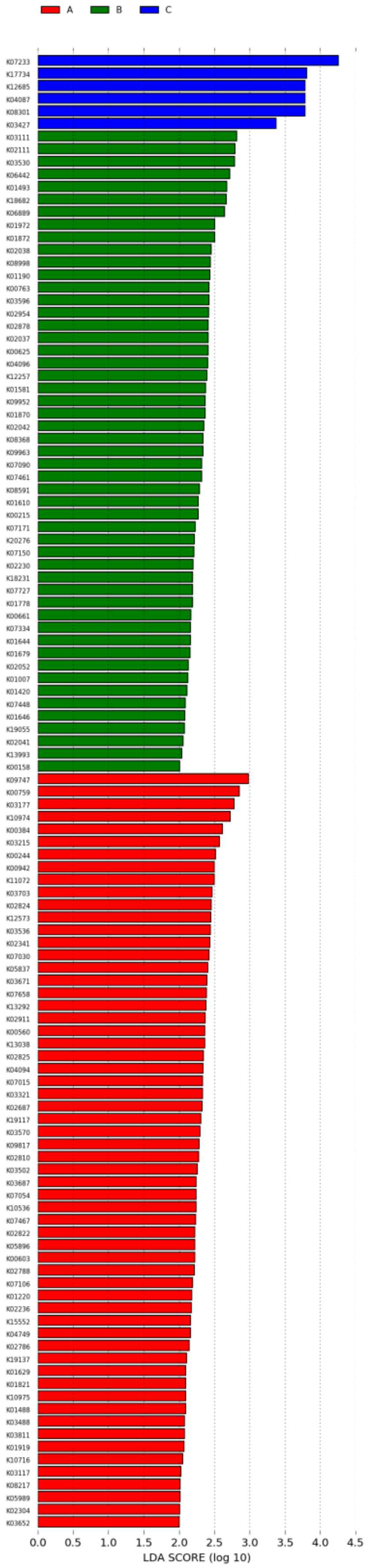

Figure S5. Comparison of KOs using LEfSe to identify differentially abundant KEGG pathway (logarithmic LDA score > 2.00, alpha value <0.05). A: Normal; B: CIN2/3; C: CX CAN.

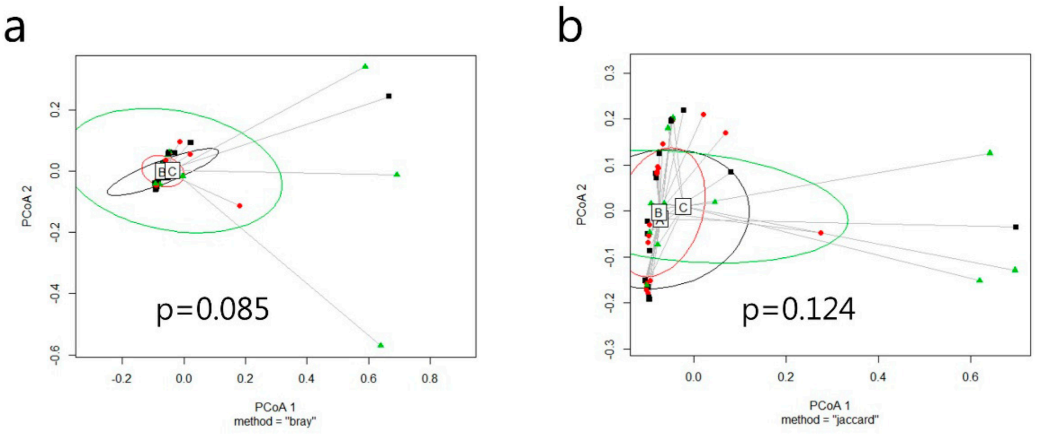

Figure S6. PCoA in KEGG pathway. (**a**) Bray-Curtis p = 0.085, (**b**) Jaccard p = 0.124. A: Normal, B: CIN 2/3, C: CX CAN.

Table S1. Mean cervical microbial abundances in 31 phyla.

Bacteria	Normal	CIN 2/3	Cervical Cancer	*p* Value [a]
Spirochaetes	56.8514	57.8374	69.1847	0.0879
Firmicutes	13.6438	13.2018	9.0367	0.0253
Proteobacteria	9.5388	9.2061	6.9775	0.1355
Actinobacteria	4.8086	5.3441	2.3254	0.5233
Bacteroidetes	5.0703	4.2996	3.0699	0.2866
Fusobacteria	2.2548	1.9275	1.9060	0.6237
Thermotogae	1.8426	2.0249	2.1575	0.0669
Tenericutes	1.8722	2.0951	1.3798	0.1001
Aquificae	1.1583	1.1769	1.2629	0.5472
Cyanobacteria	0.8963	0.9024	0.8707	0.8480
Verrucomicrobia	0.3617	0.3550	0.3331	0.4303
Chlamydiae	0.3514	0.3150	0.3301	0.7656
Synergistetes	0.2046	0.2364	0.2241	0.3898
Thermodesulfobacteria	0.1896	0.1719	0.1786	0.4731
Nitrospirae	0.1668	0.1530	0.1562	0.7061
Dictyoglomi	0.1469	0.1569	0.1513	0.8622
Bacteria (phylum)	0.1401	0.1307	0.1393	0.4846
Deinococcus.Thermus	0.1025	0.0879	0.1150	0.3708
Deferribacteres	0.0865	0.0938	0.0951	0.7152
Chlorobi	0.1264	0.0835	0.0166	0.4613
Caldiserica	0.0411	0.0517	0.0503	0.1126
Elusimicrobia	0.0363	0.0428	0.0161	0.3923
Fibrobacteres	0.0365	0.0343	0.0032	0.2340
Ignavibacteriae	0.0306	0.0290	0.0070	0.0929
Chloroflexi	0.0109	0.0168	0.0062	0.3351
Acidobacteria	0.0143	0.0119	0.0024	0.0961
Planctomycetes	0.0088	0.0057	0	0.0281
Chrysiogenetes	0.0040	0.0037	0.003	0.7979
Candidatus.Cloacimonetes	0.0031	0.0034	0.0012	0.3365
Armatimonadetes	0.0006	0.0008	0	0.2764
Gemmatimonadetes	0.0002	0	0	0.4468

[a] *p* value calculated by *Kruskal-Wallis* test.

Table S2. Mean relative abundances of COG categories.

	COG Category	Normal	CIN2/3	CX CAN	*p* Value [a]
**Cellular Processes and Signaling**	[D] Cell-cycle control, cell division, chromosome partitioning	6.0426	6.22	7.6316	0.0248
	[M] Cell wall/membrane /envelope biogenesis	3.1453	2.7111	1.3428	0.0163
	[N] Cell motility	0.3279	0.3078	0.1235	0.0127
	[O] Post-translational modification, protein turnover, and chaperones	29.4631	31.9494	42.5871	0.0136
	[T] Signal transduction mechanisms	1.8362	1.593	0.7484	0.0092
	[U] Intracellular trafficking, secretion, and vesicular transport	0.9884	0.8664	0.3831	0.0329
	[V] Defense mechanisms	2.0198	1.7914	0.8264	0.0056
	[W] Extracellular structures	0.3594	0.3025	0.1304	0.1264
	[Z] Cytoskeleton	4.6924	4.9817	6.8992	0.0233
Information Storage and Processing	[A] RNA processing and modification	0.0092	0.0086	0.0039	0.3361
	[B] Chromatin structure and dynamics	0.0100	0.0086	0.0038	0.2361
	[J] Translation, ribosomal structure and biogenesis	5.6903	5.0348	2.4959	0.0100
	[K] Transcription	2.9396	2.553	1.2190	0.0090
	[L] Replication, recombination and repair	3.6321	3.3744	1.6283	0.0216
Metabolism	[C] Energy production and conversion	1.5859	1.4063	0.6824	0.0378
	[E] Amino acid transport and metabolism	3.4002	2.9632	1.4034	0.0135
	[F] Nucleotide transport and metabolism	2.1853	1.9073	0.9373	0.0124
	[G] Carbohydrate transport and metabolism	17.873	19.3103	23.6183	0.0124
	[H] Coenzyme transport and metabolism	1.8532	1.6196	0.7515	0.0225
	[I] Lipid transport and metabolism	1.3056	1.1586	0.5345	0.0156
	[P] Inorganic ion transport and metabolism	2.2819	2.0966	1.0155	0.0200
	[Q] Secondary metabolite biosynthesis, transport, and catabolism	0.3018	0.2531	0.1200	0.0493
Poorly Characterized	[R] General function prediction only	4.0984	3.7243	2.3700	0.0111
	[S] Function unknown	2.8867	2.7175	2.0507	0.0187
Mobile Elements	[X] Mobilome: prophages, transposons	1.0717	1.1404	0.4930	0.0198

[a] *p* value calculated by *Kruskal-Wallis* test.

© 2019 by the authors. Licensee MDPI, Basel, Switzerland. This article is an open access article distributed under the terms and conditions of the Creative Commons Attribution (CC BY) license (http://creativecommons.org/licenses/by/4.0/).
